# Anti-angiogenic and anti-proliferative activity of ziziphus leaf extract as a novel potential therapeutic agent for reducing hepatic injury in experimental hamster schistosomiasis

**DOI:** 10.1371/journal.pntd.0011426

**Published:** 2023-06-20

**Authors:** Thamer Alghamdi, Doaa A. Salem, Mohamed F. El-Refaei

**Affiliations:** 1 Department of Surgery, Division of Hepatobiliary Surgery, Faculty of Medicine, Al-Baha University, Al-Baha, Saudi Arabia; 2 Department of Medical Parasitology, Faculty of Medicine, Al-Baha University, Al-Baha, Saudi Arabia; 3 Faculty of Medicine, Mansoura University, Mansoura, Egypt; 4 Department of Medical Biochemistry, Faculty of Medicine, Al-Baha University, Al-Baha Saudi Arabia; 5 Genetic Institute, Sadat City University, Sadat City, Egypt; George Washington University, UNITED STATES

## Abstract

**Background:**

Schistosomiasis is one of the most prevalent helminthic infections worldwide. Praziquantel (PZQ) resistance poses a possible danger to the disease’s ability to be controlled. Little is known about the role of *Ziziphus spina-christi* leaf extract (ZLE) in the treatment of hepatic schistosomiasis. However, no study has explored ZLE’s anti-angiogenic and anti-proliferative activity as a possible mechanism for reducing hepatic injury in this context. Therefore, this study aimed to evaluate the therapeutic potential of ZLE as an anti-angiogenic, and anti-proliferative agent in hamsters infected with *S*. *mansoni*.

**Methods:**

Fifty hamsters were used and divided into 5 groups (10 hamsters each); noninfected untreated (controls), noninfected treated with ZLE, infected untreated, infected treated with PZQ- and infected treated with ZLE. Anti-angiogenic and anti-fibrotic effects of the drugs were assessed pathologically through the immunohistochemical expression of VEGF, Ki-67, and TGF β1 in liver sections. Some oxidative stress parameters were measured in hepatic homogenates (NO, GSH, GST, and SOD), and serum liver enzymes were also assessed.

**Results:**

A significant decrease in worm burden, granuloma size, granuloma area, and numbers in the ZLE- and PZQ-treated groups compared to the infected untreated group, and the decrease in granulomas number and tissue egg load was significantly lower in PZQ treated group compared to ZLE treated group (p<0.05). ZLE exhibited significant anti-angiogenic and anti-fibrotic effects on granulomas, illustrated by significantly lower expression of VEGF and TGF-β1 than infected untreated and PZQ-treated groups. ZLE exhibits antiproliferative activity evidenced by a significant reduction of positive Ki-67 hepatocytes percentage compared to the infected untreated group. Moreover, ZLE exhibits potent antioxidant effects evidenced by a significantly lowered NO and conservation of hepatic GSH, GST, and SOD in hepatic homogenates compared to infected untreated and PZQ-treated groups (p<0.05).

**Conclusion:**

Our results point to ZLE as a promising hepatoprotective therapeutic tool in the treatment of schistosome hepatic fibrosis as it has anti-angiogenic, anti-proliferative, anti-fibrotic, and antioxidant effects in hamsters infected with *S*. *mansoni*, providing scientific support for its use in conventional medicine.

## Author summary

Eggs hepatic granuloma and fibrosis are the predominant pathological features of hepatosplenic schistosomiasis. This pathology can cause death from portal hypertension and ascites brought on by hepatic fibrosis. Soluble egg antigens also can trigger hepatocellular carcinogenesis by stimulating proliferation. Angiogenesis plays a crucial part in developing and progressing hepatic fibroproliferative and ischemic disorders. We aimed to evaluate the therapeutic potential of ZLE as an anti-angiogenic, and anti-proliferative agent as a promising hepatoprotective therapy against *S*. *mansoni-*induced liver fibrosis in hamsters. Our results revealed that significant decrease in granuloma size and numbers in the ZLE- and PZQ-treated groups compared to the infected untreated group and the decrease in granulomas number and tissue egg load were significantly lower in PZQ treated group compared to ZLE treated group. ZLE exhibited significant anti-angiogenic and anti-fibrotic effects on granulomas, illustrated by significantly lower expression of VEGF and TGF-β1 than infected untreated and PZQ-treated groups. ZLE exhibits antiproliferative activity evidenced by a significant reduction of positive Ki-67 hepatocytes percentage compared to the infected untreated group. Moreover, ZLE exhibits potent antioxidant effects evidenced by a significantly lowered NO and conservation of hepatic GSH, GST, and SOD in hepatic homogenates compared to infected untreated and PZQ-treated groups.

## 1. Introduction

Schistosomiasis is still one of the most common helminthic illnesses in the world and is categorized as a neglected tropical disease of global medical concern. More than 700 million people reside in endemic areas, and it is believed that more than 240 million people are currently infected worldwide [1].

Hepatic granuloma and fibrosis brought on by worm eggs are the predominant pathological features of hepatosplenic schistosomiasis. Patients with hepatosplenic schistosomiasis typically die from portal hypertension and ascites brought on by hepatic fibrosis [2].

The progression of schistosomiasis can be slowed down by reducing granuloma formation and the start of fibrosis caused by schistosome eggs. Determining the essential chemicals that trigger and control hepatic granuloma and fibrosis during Schistosoma infection is crucial [3].

According to growing data, angiogenesis plays a crucial part in developing and progressing hepatic fibroproliferative and ischemic disorders. Inflammation, hepatic stellate cells (HSCs), and hypoxia are significant inducers of angiogenesis. [4].

Experimental models and human research have both described schistosome-induced angiogenesis. Inducing endothelial proliferation has been linked to living eggs and soluble egg antigens (SEA), which may encourage angiogenesis within hepatic granulomas by increasing endothelial cell vascular endothelial growth factor (VEGF). The neovascularization that has been observed may also be influenced by the conditions brought on by artery occlusions, such as hypoxia, an acidic pH, and low glucose levels [5].

Ki-67 is a nuclear protein expressed only in the proliferating cells therefore it is widely used as a marker to assess active cell proliferation in normal and tumor cell populations. Even though Ki-67 isn’t essential for cell proliferation, recent studies raise the question of whether Ki-67 plays a role in tumorigenesis. Since Ki-67 is an intrinsically disordered protein without an inherent enzyme, therapeutic targeting of Ki-67 will likely be difficult. [6,7].

An evaluation of the Ki-67 hepatocyte proliferative index may be useful in identifying cirrhotic patients who are at risk for hepatocellular carcinoma, as well as for analyzing liver regeneration and carcinogenesis processes in the liver [8].

Schistosome infections cannot currently be prevented by vaccination. Anti-schistosomal medications effectively eliminate mature worms and halt the production of schistosome eggs, but they are unable to repair liver fibrosis that has already occurred, especially in chronic and severe phases of schistosomiasis. Therefore, it is still challenging to identify a cure for liver fibrosis brought on by schistosomiasis. It has been demonstrated that praziquantel (PZQ) effectively treats liver fibrosis and schistosomiasis. Recent drug resistance and chemical toxicity reports from PZQ have sparked interest in developing innovative, reasonably priced, safe medications that protect the hepatic organs through localized plant therapies with antiparasitic properties [9–11].

The Rhamnaceae family includes the wild tree *Ziziphus spina-christi* (ZSC), whose fruits are frequently used in traditional medicine and are widely available in Al-Baha, Saudi Arabia. A small, orange-yellow fruit is produced by this tropical evergreen tree. In Middle Eastern nations, the untamed plants are known as sidr [12].

Since there is very little research about the effect of ZSC on schistosomal hepatic fibrosis, our study is the first study that explored the anti-angiogenic and anti-proliferative activity of ZLE as a possible mechanism for reducing fibrosis in this context.

We aimed to evaluate the therapeutic potential of ZLE as an anti-angiogenic, and anti-proliferative agent as a promising hepatoprotective therapy against *S*. *mansoni-*induced liver fibrosis in hamsters.

## 2. Material and methods

### 2.1 Ethics statement

Laboratory animals were handled according to the guidelines for the care and use of laboratory animals outlined by the institution and the national guide. The study protocol was approved by the Ethics Committee Board of the Faculty of Medicine at Al-Baha University, Kingdome of Saudi Arabia (REC/SUR/BU-FM/2022/59).

### 2.2 Animal model

Fifty golden hamsters, weighing 115 to 130 g were included in this study. The resource equation’ approach by Arifin and Zahiruddin was applied to calculate the sample size in this study [13]. Based on Smithers and Terry, the hamsters were infected percutaneously with *S mansoni* after being anesthetized, abdomen-shaved, and then exposed percutaneously to a suspension containing approximately 250 living cercariae in the metal ring using a micropipette over 30 minutes [14].

The hamsters were maintained on a standard laboratory diet and water in the animal house under controlled temperature at 20 to 22°C and 12 hours of dark/light cycle.

The hamsters were allocated to five groups of ten each noninfected untreated (controls) group (I), noninfected treated with ZLE, group (II) infected untreated group, (group III) infected treated with PZQ (reference drug) (group IV), and infected treated with ZLE alone (group V).

Forty-nine days after exposure to cercariae, hamsters in each group were administrated the drug doses orally for all treatments as scheduled and euthanized nine weeks post-infection and were dissected.

Appropriate anesthetic and euthanizing procedures were used to ensure that animals did not suffer at any stage of the experiments, according to the legal ethical guidelines of our Ethical Committee which follow the American Veterinary Medical Association (AVMA) Guidelines for the Euthanasia of Animals. Overdose intraperitoneal anesthesia with thiopental sodium was used for euthanasia, and the hamster was euthanized after the blood was removed for testing [15].

#### Drugs and dosage

**Praziquantel (PZQ) suspension preparation.** PZQ 600 mg/tablet (Distocide; EIPICO. Pharmaceuticals, Egypt) is powdered, suspended in 70% glycerin, and given as a freshly prepared by gavage 100 μL of 500 mg/kg for two consecutive days [16].

***Ziziphus spina-christ* leaf extract (ZLE) preparation.** ZLE were prepared according to Hafiz and Mubaraki [17]. An expert taxonomist from the Botany Department, Faculty of Science, Al-Baha University selected and verified the *Ziziphus spina-christ* leaves, which were washed and air-dried in the shade. After grinding the dried ZSC leaves finely, they were immersed in 70% (v/v) methanol for 48 hours at 4°C. A rotary evaporator at 45°C was utilized to evaporate the extract to a semi-dry state and then liquefy it in distilled water. As a result, ZLE was obtained and stored at -20°C. At 49 days post-infection, ZLE treatment was started by gavage with 100 μL of 400 mg/kg ZLE daily for 10 consecutive days according to [18].

Hamsters were used to assess ZLE’s acute oral toxicity. After fasting for 12 hours, the animals were given a single dose of extracts dissolved in distilled water and monitored for mortality over the next 48 hours (short-term toxicity). Following OECD guidelines for short-term toxicity, the next animal’s dose was determined [19].

### 2.3 Assessment of parasitological criteria

#### 2.3.a Worm recovery

Hamsters were subjected to a hepatic-porto-mesenteric perfusion procedure to collect adult *S*. *mansoni*, worm burdens were counted, and the percentage of change in total worms was calculated [20].

#### 2.3.b. Ova counts in feces and hepatic tissue egg load

Feces were collected one week before treatment administration to microscopically confirm the presence of parasite eggs and again one week after treatment to ensure that treatment was effective.

The hepatic tissue egg load was performed using defined sections of the liver of each hamster. Cheever’s method was used to count the number of ova per gram of tissue [21]. The hamster liver was digested overnight at 37°C in 4% potassium hydroxide. Following digestion, tissue suspensions were centrifuged at 1500 rpm for five minutes to remove supernatants. The number of eggs was determined in two aliquots of 100 L each after three cycles of washing and centrifugation using a light microscope. The results were represented as the mean number of eggs per gram of liver tissue [21].

#### 2.3.c. Histopathology and granulomas measurement

In all groups, liver tissue samples were fixed with 10% neutral buffered formalin immediately for 24 hours and embedded in paraffin after dehydration with increasing concentrations of ethanol. Sections of 5 μm thickness were deparaffinized, and stained with hematoxylin and eosin, before being examined under an Olympus light microscope. The following characteristics were measured for morphometric analysis of the granulomas: area and mean granuloma diameter. Moreover, the associated histopathological changes were evaluated using stained sections. The diameter of granulomas containing a single egg in the center was measured Using a calibrated ocular micrometer. The mean granuloma diameter was calculated by measuring two diameters of the lesion at right angles to each other. At a minimum, the diameters of up to 50 egg-induced granulomas were determined per animal on an ocular micrometer, 100 granulomas for each group were examined at 100× magnification, and the mean size ± standard deviation was given in μm. For the area of granuloma, images were taken with an analog camera (Sony, 640 x 480 pixels, RGB) and transmitted to a computer running image analysis software (Scion Image; Scion).

#### 2.3.d. Histological activity index assessment

Based on a modified quantitative Ishak scoring system, scores of 1–3 corresponded to minimal liver damage, scores of 4–8 to mild, scores of 9–12 to moderate, and scores of 13–18 to severe liver damage [22].

### 2.4. Oxidative stress markers assessment

Known liver tissue weights were homogenized in a cold buffer containing 250 mM sucrose, 10 mM MgCl_2_, and 50 mM Tris-HCl with pH 7.4. A 10% of liver homogenates were used for some oxidant/antioxidant assessment. Using Ellman’s method, reduced glutathione (GSH) level in liver homogenates was measured [23]. The activity of superoxide dismutase (SOD) was measured by Nishikimi et al. on tissue homogenates to assess antioxidant enzyme activity [24], and glutathione-S-transferase (GST) was assayed according to Habig et al. [25]. The nitric oxide (NO) was assayed according to the method of Green et al. [26].

### 2.5 Liver function tests assessments

Immediately after hamster euthanization, whole blood was obtained by cardiac puncture and collected in plain tubes and serum was obtained by centrifugation at 4000 rpm for 10 min and preserved at -20°C until used for liver function tests assessments. Aspartate aminotransferase (AST), alanine aminotransferase (ALT), and alkaline phosphatase (ALP) activity were estimated using available commercial kits provided by SIGMA ALDRICH (MAK055-IKT, MAK052-IKT, and SCR-004, respectively) according to manufactures protocol.

### 2.6 Immunohistochemistry of liver specimens

Immunohistochemical staining of TGF-β, VEGF, and Ki-67 was performed on 4–5-micron thick tissue sections obtained from paraffin-embedded tissue. After deparaffinization by using xylene and rehydrated in descending grades of ethanol. Endogenous peroxidase activity was blocked using 6% hydrogen peroxide for 30 minutes. Sections were incubated with a primary antibody against TGF-β1 (Recombinant Rabbit Monoclonal Antibody (PD00-17) (Thermo Fisher, Catalog # MA5-44667, 1:200 dilution), VEGF (Recombinant Rabbit Monoclonal Antibody (SP07-01) (Thermos Fisher, Catalog # MA5-32038, 1:200 dilution), and Ki-67 (Recombinant Rabbit Monoclonal Antibody (SP6) Catalog Number] (Thermo Fisher, Catalog # MA5-14520, 1:200 dilution) overnight at 4°C. After that, the secondary antibody (Goat anti-Rabbit IgG (H+L) HRP) (Thermos Fisher, Catalog # 31460) was used and incubated for 30 minutes. Staining kits were used according to the manufacturer’s instructions (Thermo Fisher Scientific). Afterward, the sections were counterstained with hematoxylin and examined under a microscope.

To examine the liver sections, a Zeiss light microscope (Oberkochen, Germany) was used. Semi-quantitative analyses were performed for TGF-β1, and VEGF on three randomly selected fields from five different immunohistochemically-stained specimens to determine the color intensity. Based on the intensity, the result was expressed as + (weak immunoreactivity), ++ (moderate immunoreactivity), +++ (strong immunoreactivity), or ++++ (very strong immunoreactivity).

Nuclear staining was defined as the presence of a Ki-67 positive cellular index. All stained nuclei, regardless of staining intensity, were rated as positive. In five randomly chosen fields, a typical light microscope was used to count cells at 100 x magnification. By counting 1,000 cells on each slide, it was possible to calculate the percentage of cells that express Ki-67. A qualified pathologist analyzed Ki-67-labeled cell counts using the following formula: Number of IHC-positive cells (KI-67) *100/Total number of cells observed [27].

### 2.7 Statistical analysis

The statistical analysis was carried out using the statistical software program SPSS version 21 (IBM, Chicago, IL, USA). The data are expressed as mean ± SD. The distribution of tested variables was examined with the Shapiro–Wilk test for normality. The Student’s t-test was used to compare the means between the two groups. The one-way analysis of variance (for normally distributed data) or the Kruskal-Wallis’s test (for nonnormally distributed data) was employed for the comparison of three or more groups, followed by the Bonferroni post hoc test. P < 0.05 was considered statistically significant.

## 3. Results

### 3.1. Assessment of parasitological criteria

The parasitological burden (hepatic tissue egg load, number, size of granulomas, and granuloma area) in the hepatic tissues of infected hamsters with *S*. *mansoni* was evaluated.

#### 3.1.a. Hepatic tissue egg load, and the number of granulomas

**Fig 1:**
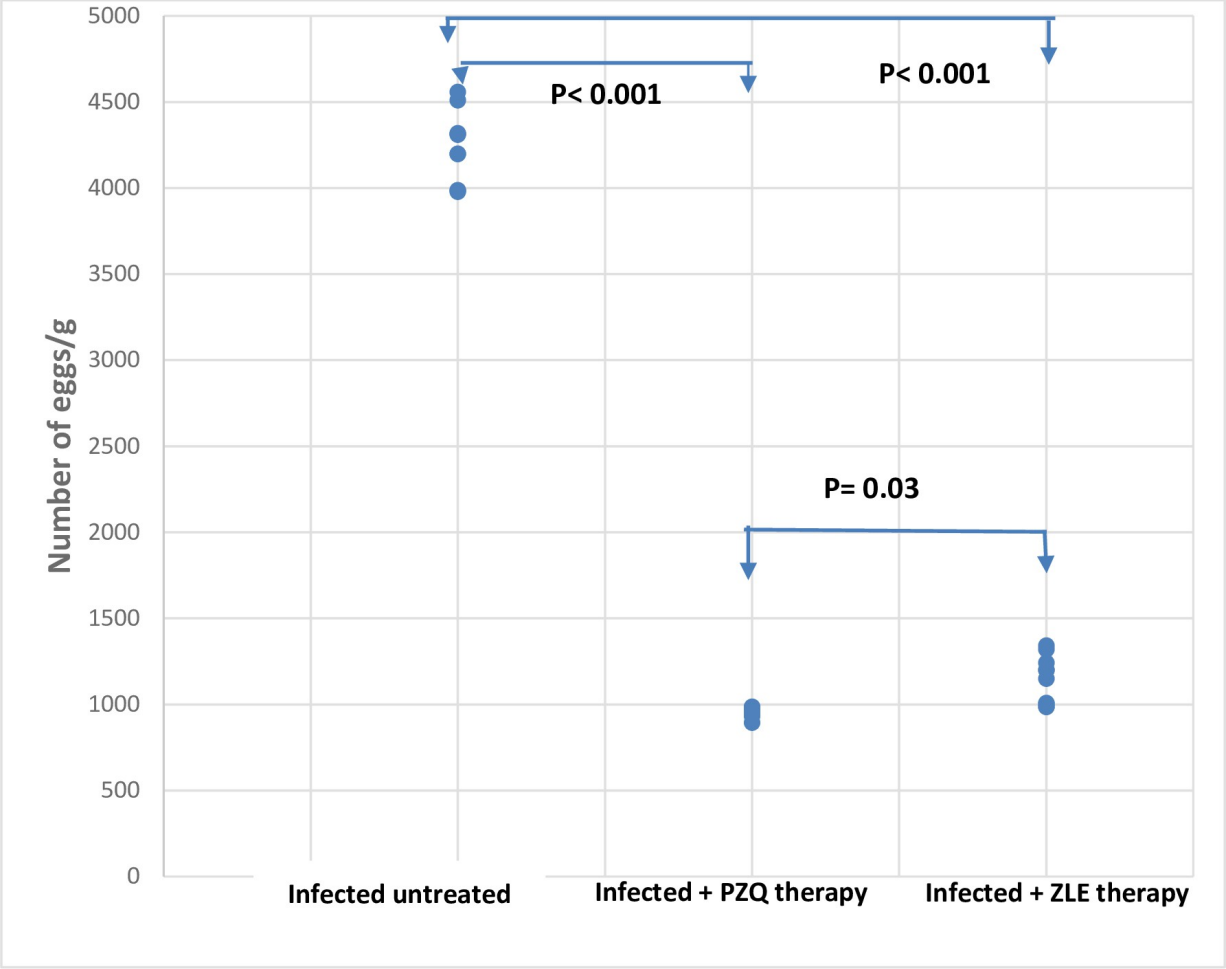
Dispersion graph illustrating a significant reduction of the number of hepatic tissue egg load with PZQ and ZLE therapy compared to infected untreated hamsters with *S*. *mansoni* (p< 0.001 for both). However, PZQ therapy significantly reduced the hepatic tissue egg load compared to ZLE therapy (p = 0.03). The dispersion graph data of 10 hamsters/group. PZQ, praziquantel; ZLE, Ziziphus leaf extract.

There was a significant reduction in the hepatic tissue egg load of the infected hamsters treated with PZQ by 77.3% (4237.5±208.5 vs 961±28.3, p < 0.001) and the ZLE-treated group by 73% (4237.5±208.5 vs. 1144.6±138.6, p < 0.001) compared to the infected untreated group. When compared to the ZLE-treated group, the PZQ regimen significantly reduced the hepatic tissue egg load (p = 0.03) (Table 1 and Fig 1).

**Table 1:** The effect of ZLE and Praziquantel treatment on hepatic tissue egg load, number, diameters of granulomas, and granuloma area among infected groups.

Group	Hepatic tissue egg load	Number of granulomas	Diameter of granulomas	Granuloma area
**Infected untreated**	4237.5±208.5	4.4±1.1	546.5. ±7.7	51.4±7.4x10^3^
**Infected + PZQ therapy**	961±28.1	2.1±0.7	314.4±13.8	40.6±9.1X10^3^
**Percent of changes**	77.3%	52.3%	42.5%	21%
**P1**	**< 0.001**	**< 0.001**	**< 0.001**	**0.001**
**Infected +ZLE therapy**	1144.6±138.6	3.2±0.6	246.4±8	32.5±3.7 X 10^3^
**Percent of changes**	73%	29.5%	54.9%	36.8%
**P2**	**<0.001**	**0.007**	**< 0.001**	**< 0.001**
**P3**	**0.03**	**0.002**	**< 0.001**	**0.02**

P1 between infected untreated and infected + PZQ therapy, p2 between infected untreated and infected + ZLE therapy, p3 between infected + PZQ therapy and infected +ZLE therapy

**Fig 2:**
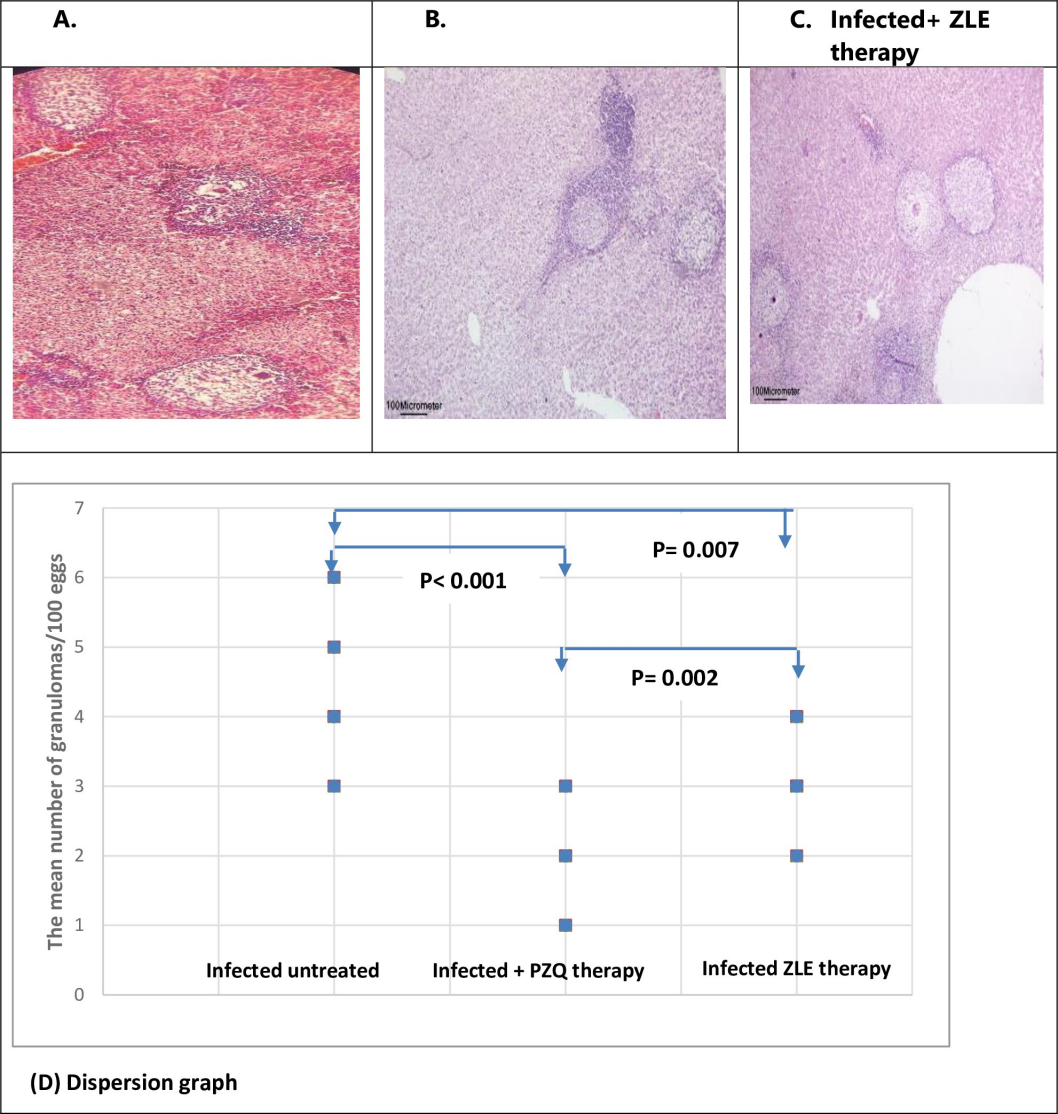
(A) The Liver histopathology section stained with H&E shows egg granulomas in infected untreated hamsters. (B) The Liver histopathology section stained with H&E shows egg granulomas in infected hamsters treated with PZQ. (C) The Liver histopathology section stained with H&E shows egg granulomas in infected hamsters treated with ZLE. (D) PZQ and ZLE therapy significantly reduces the mean number of granulomas in hamsters infected with *S*. *mansoni* compared to the infected untreated group (p < 0.001, 0.007, respectively). This reduction in granuloma number was significantly lower among the PZQ regimen compared to the ZLE regimen (p = 0.002). The dispersion graph data of 10 hamsters/group. PZQ, praziquantel; ZLE, Ziziphus leaf extract.

The mean number of granulomas was 4.4±1.1 in infected untreated livers, the PZQ treatment regimen and ZLE treatment significantly reduced their numbers to 2.1±0.7 and 3.2±0.6 respectively. (p < 0.001, 0.007, respectively). This reduction in granuloma number was significantly lower among the PZQ regimen compared to the ZLE regimen (p = 0.002). (Table 1 and Fig 2). Therefore, PZQ therapy exhibits a more potent anti-schistosomal effect than ZLE therapy evidenced by more reduction in both hepatic tissue egg load, and the number of granulomas.

#### 3.1.b. Diameter of granulomas and granuloma area

**Fig 3:**
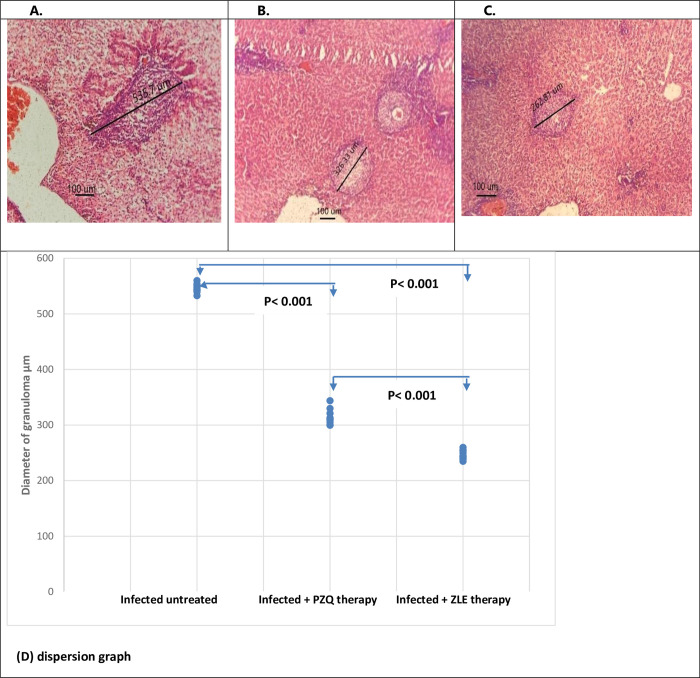
A) The Liver histopathology section stained with H&E shows egg granuloma diameter in infected untreated hamsters. B) The Liver histopathology section stained with H&E shows egg granuloma diameter in infected hamsters treated with PZQ. C) The Liver histopathology section stained with H&E shows egg granuloma diameter in infected hamsters treated with ZLE. (D) PZQ and ZLE therapy significantly reduces the mean diameter of hepatic granulomas in hamsters infected with *S*. *mansoni* compared to the infected untreated group (p < 0.001 in both cases) with more reduction with ZLE therapy (p. < 0.001). The dispersion graph data of 10 hamsters/group. PZQ, praziquantel; ZLE, Ziziphus leaf extract.

There was a highly significant reduction in the mean diameter of granulomas in PZQ and ZLE- treated hamsters (314.4±13.8, 246.4±8 μm respectively) compared to the infected untreated group (546.5. ±7.7 μm) (p < 0.001 in both cases). When compared to the PZQ-treated group, the ZLE- treated hamsters had a much more reduction in hepatic granuloma size by 21.6% (p. < 0.001). In addition, the mean granuloma area in hamsters treated with both PZQ and ZLE was significantly reduced by 21% and 36.8% respectively compared to infected untreated hamsters (40.6±9.1X10^3^ μm^2^, 32.5±3.7 X 10^3^ μm^2^ VS 51.4±7.4x10^3^ μm^2^, p = 0.001, < 0.001 respectively) (Table 1 and Figs 3 and S1). Therefore, ZLE therapy exhibits a more potent effect on granuloma size and granuloma area compared to PZQ therapy.

#### 3.1.c. Total worm recovery

The mean number of worms recovered from the infected untreated group was 19.8± 1.9. Treatment of infected hamsters with PZQ and ZLE resulted in a reduction in the mean number of worms of 0.8±0.3, 7.6 ± 1.1 respectively (the reduction percentage is 96% and 61.6% respectively) (p < 0.001 for all) (S2 Fig).

### 3.2 Histopathology parameters

#### 3.2.a. Histological liver activity index (HAI)

**Fig 4:**
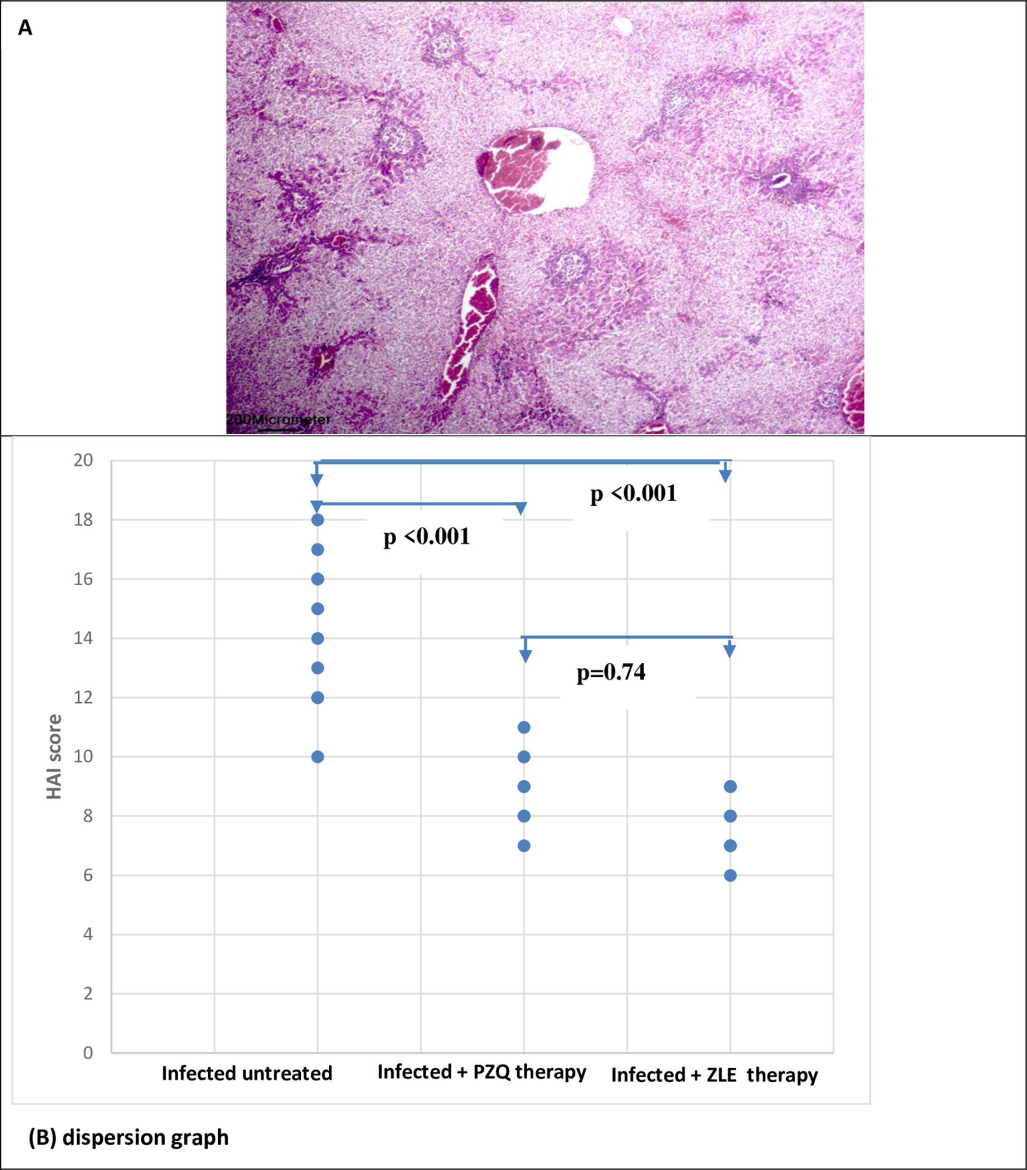
A) The Liver histopathology section stained with H&E section shows a congested central vein and egg granulomas in infected untreated hamsters. B) The histologic activity index (HAI) score was significantly reduced with PZQ and ZLE therapy in hamsters infected with S. mansoni (p< 0.001 for both). HAI score was categorized from 12–18 for the infected livers. The dispersion graph data of 10 hamsters/group.

*S*. *mansoni* infection in hamsters generated a significant granulomatous inflammatory response in the liver, as evidenced by infiltration with inflammatory cells, vacuolization of the cytoplasm, and hepatocyte degradation. Trapped eggs encircled with concentric fibrosis with many fibroblasts, is a characteristic of granulomas. Disorganization of the liver architecture due to the presence of many granulomas. The hepatic sinusoids were also enlarged and appeared to have more Kupffer cells. Treatment with ZLE and PZQ significantly reduces the histological liver activity index (p <0.001 in both groups). HAI was categorized from 12–18 for the infected livers in comparison to 0–1 for noninfected controls. The mean HAI was 14.3±2.5 in the infected untreated group compared to 8.9±1.2 in the PZQ-treated group and 7.8± 1.03 in the ZLE-treated group (Table 2 and Fig 4).

**Table 2:** Effect of ZLE therapy on histopathological changes in hepatic tissues of noninfected and infected hamsters with *S*. *mansoni*.

Group	Microscopic parameters					
	Necrosis or apoptosis	Hemorrhage	Disorganized sinusoid	Infiltration of lymphocyte	Hyperplasia of Kupffer cell	Hepatocyte swelling
**Noninfected untreated**	0	0	0	0	0	0
**Noninfected+ ZLE therapy**	0	0	0	0	0	0
**Infected untreated**	+++	++	++	+++	+++	++
**Infected + PZQ therapy**	+	+	+	+++	+	+
**Infected +ZLE therapy**	+	+	+	+	0	+

0: absent; +: mild; ++; moderate; and +++: severe.

#### 3.2.b. Immunohistochemical aspects

VEGF and TGF-β1 immunohistochemistry expression in hamsters’ hepatic tissues were used to evaluate the antiangiogenic and anti-fibrotic effect pathologically. Monoclonal antibodies to both VEGF and TGF- β1 were not expressed in the hepatic tissues of noninfected untreated hamsters (group I).

**Fig 5:**
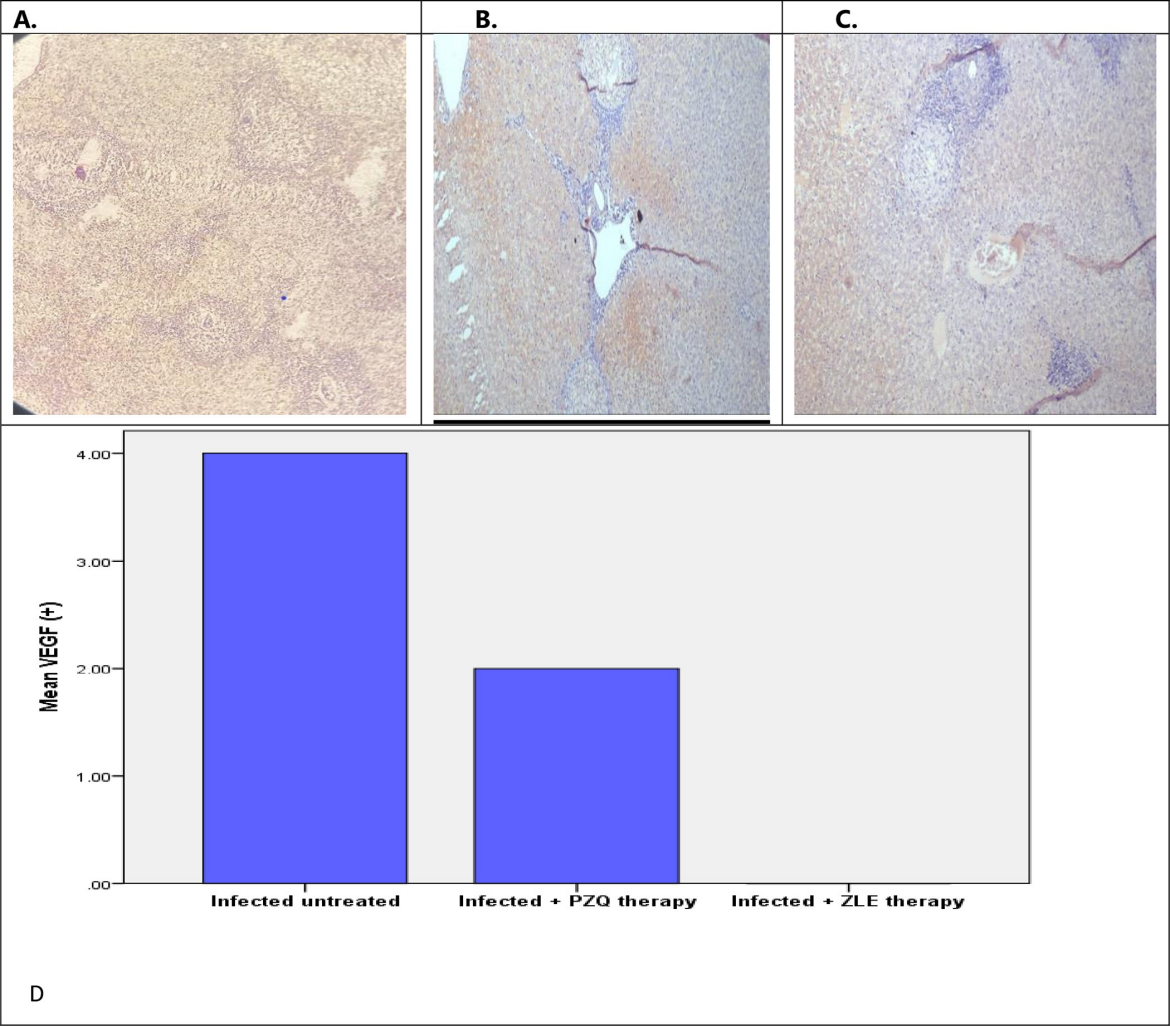
A) Immunohistochemistry for VEGF in Infected untreated hamsters shows positive VEGF highlights the endothelial cells of liver sinusoids with positive reactions within granulomas **(**x200) (++++). B) Immunohistochemistry for VEGF in infected hamsters treated with PZQ shows positive VEGF highlighting the endothelial cells of liver sinusoids with focal positive reaction within granulomas (x200) (++). C) Immunohistochemistry for VEGF in infected hamsters treated with ZLE shows positive VEGF lightening the endothelial cells of liver sinusoids with negative within granulomas (x200) (—). D) There was a significant reduction in VEGF immunohistochemical expression in the hepatic tissues of hamsters infected with *S*. *mansoni* treated with PZQ and ZEL, however, ZLE therapy caused a potent significant reduction in VEGF hepatic tissue expression (negative expression) compared to the PZQ-treated group. VEGF, vascular endothelial growth factor; PZQ, praziquantel; ZLE, Ziziphus leaf extract. -The intensity was expressed as + (weak immunoreactivity), ++ (moderate immunoreactivity), +++ (strong immunoreactivity), or ++++ (very strong immunoreactivity). -The intensity was expressed as + (weak immunoreactivity), ++ (moderate immunoreactivity), +++ (strong immunoreactivity), or ++++ (very strong immunoreactivity).

**Fig 6:**
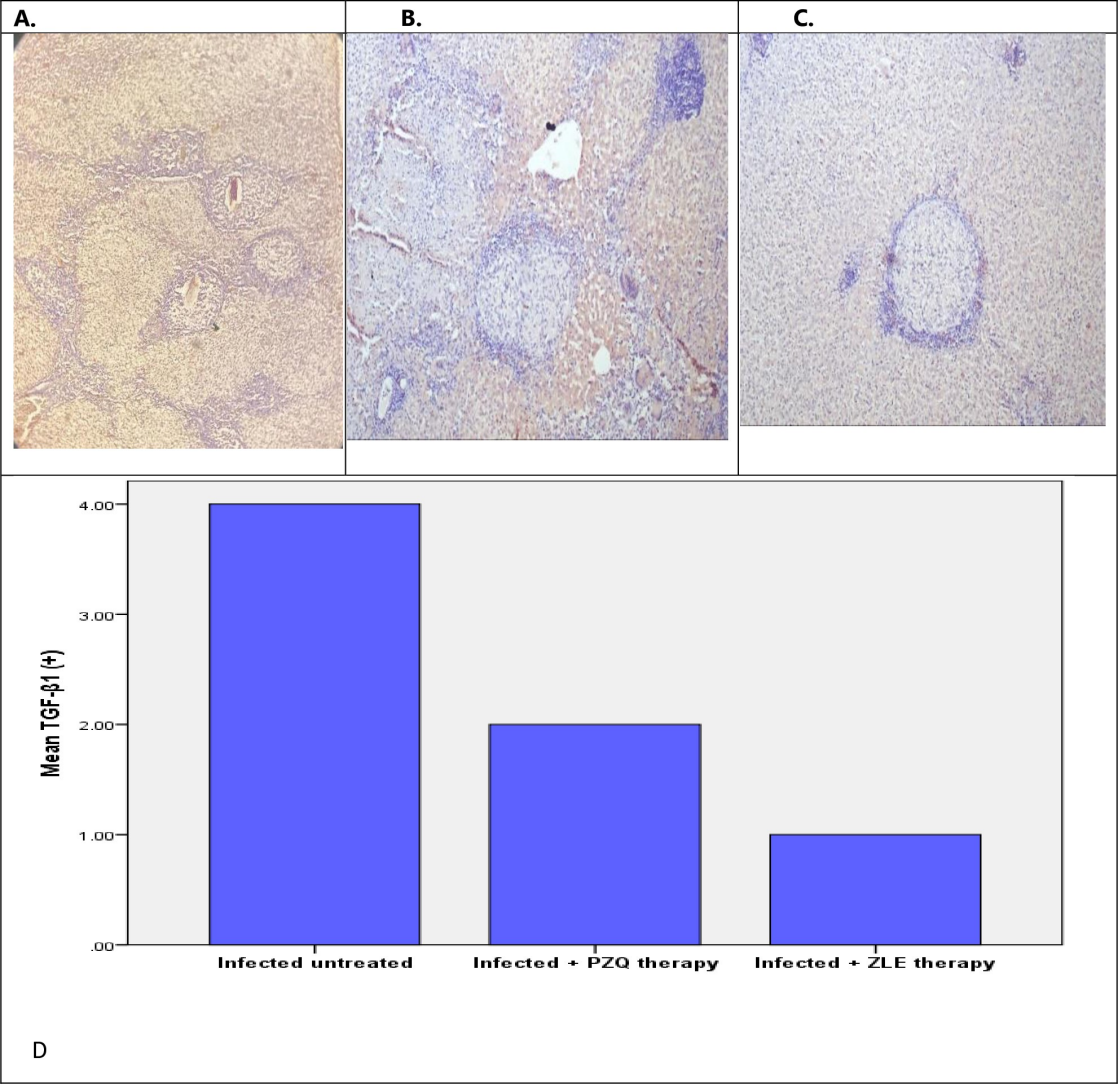
A) Immunohistochemistry for TGF-β1 in Infected untreated hamsters shows strong positive reaction within the granulomas and the fibrous capsule (x200) (++++). B) Immunohistochemistry for TGF-β1 in infected hamsters treated with PZQ shows moderate reaction within the granulomas and the fibrous capsule (x200) (++). C) Immunohistochemistry for TGF-β1 in infected hamsters treated with ZLE shows weak reaction within the granulomas and within the fibrous capsule (x200) (+). D) There was a significant reduction in TGF-β1 immunohistochemical hepatic tissues expression of hamsters infected with *S*. *manson*i treated with PZQ and ZEL, however, ZLE therapy caused a more significant reduction in TGF-β1 hepatic expression compared to the PZQ-treated group. TGF- β1, Transforming growth factor- β1; PZQ, praziquantel; ZLE, Ziziphus leaf extract. -The intensity was expressed as + (weak immunoreactivity), ++ (moderate immunoreactivity), +++ (strong immunoreactivity), or ++++ (very strong immunoreactivity).

Infected hamsters (group III) demonstrated pronounced expression of VEGF and TGF-β1 monoclonal antibodies. There was a significant reduction in VEGF expression in the liver of hamsters infected groups treated with PZQ and ZEL when compared to the infected untreated group, however, treatment with ZLE caused a potent significant reduction in VEGF hepatic expression (negative expression) compared to the PZQ-treated group (Fig 5). Moreover, treatment with PZQ and ZEL caused a significant reduction in TGF-β1 expression in the liver when compared to the infected untreated group. Therefore, both ZLE and PZQ-treated groups exhibited a significant anti-fibrotic role in granulomas as demonstrated by weak and moderate TGF- β1 expression in the granulomas respectively (Fig 6).

According to our immunohistochemistry findings, ZLE treatment has stronger anti-angiogenic and anti-fibrotic effects than PZQ treatment in hepatic granulomas and fibrosis (Figs 5 and 6).

**Fig 7:**
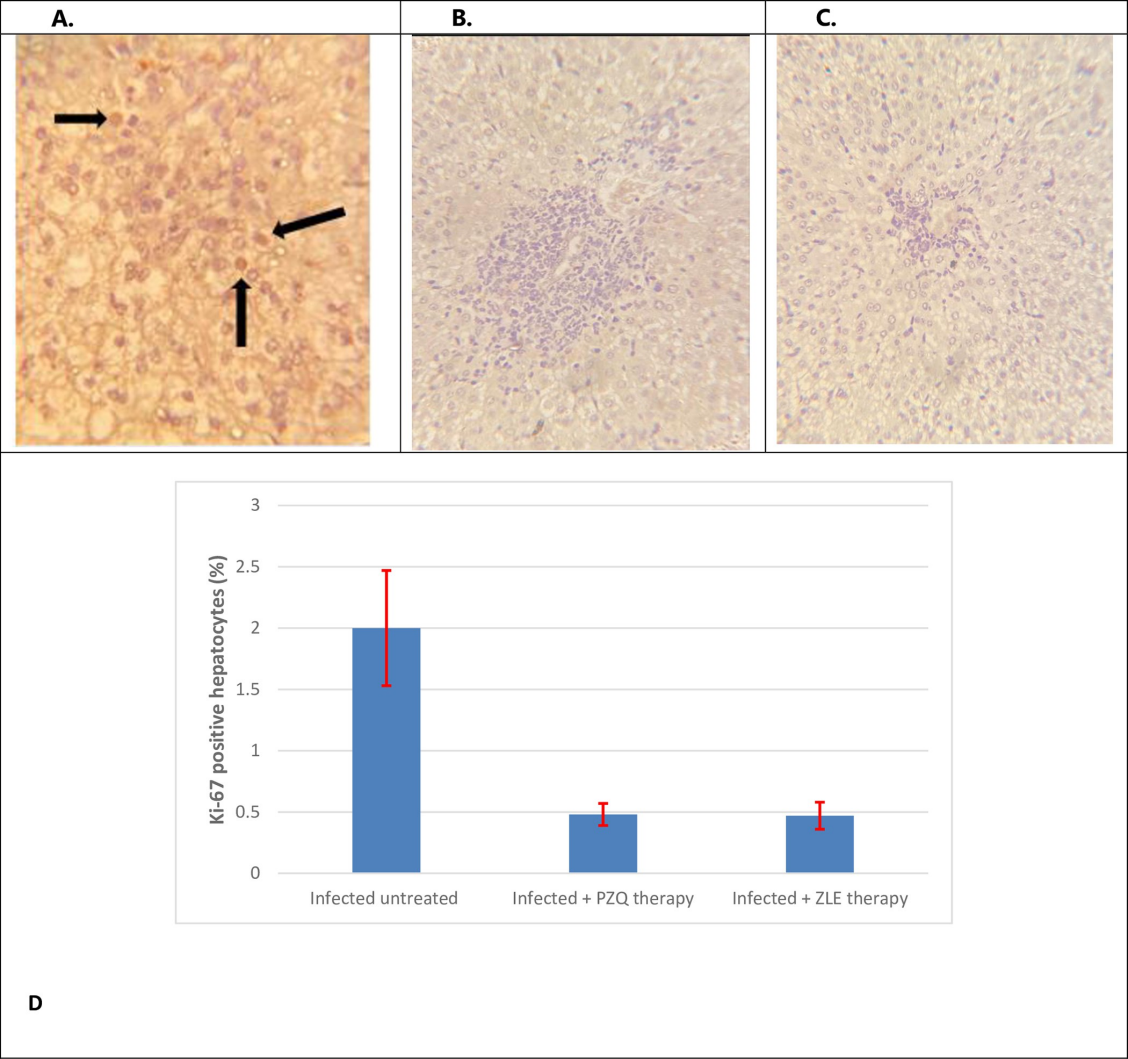
A) Immunohistochemistry for Ki-67 in infected untreated hamsters shows positive reaction (x400). B) Immunohistochemistry for Ki-67 in infected hamsters treated with PZQ the negative reaction (x400). C) Immunohistochemistry for Ki-67 in infected hamsters treated with ZLE shows the negative reaction (x400) (<1%). D) The percentage of immunohistochemical expression of Ki-67-positive hepatocytes was low. (2±0.47) in the hepatic tissue of the infected untreated group and significantly reduced 0.48±0.09) in both praziquantel treated groups(0.47±0.11) and ZLE treated group (p < 0.001). -The Ki-67 staining pattern was in the nuclei of hepatocytes in a directional pattern between granuloma and central veins. -The error bar data are mean ± SD of 10 hamsters/group. PZQ, praziquantel; ZLE, Ziziphus leaf extract.

Immunohistochemical analysis of Ki-67 showed enhanced hepatocyte proliferation in *S*. *mansoni*-infected hamsters compared to noninfected untreated hamsters. The percentage of Ki-67 positive hepatocytes was low (1–2%) in the infected untreated group and significantly reduced (less than 1%) in both Praziquantel and ZLE-treated groups. The Ki-67 staining pattern was in the nuclei of hepatocytes in a directional pattern between granuloma and central veins (Fig 7). According to these results, both PZQ and ZLE exert an anti-proliferative effect on hepatic granuloma.

### 3.3 Impact on oxidant/antioxidant parameters

Hepatic homogenates NO increased significantly in hamsters infected with *S*. *mansoni* (group III) than in control hamsters (group I) (p < 0.001). Treatment with PZQ and ZLE highly significantly reduced the NO levels in hepatic homogenates (p < 0.001 for both), however, ZLE therapy significantly reduce the NO in hepatic homogenates compared to PZQ therapy (p = 0.02) (Table 3 and S3A Fig).

**Table 3:** The effect of ZLE and Praziquantel treatment on liver homogenate glutathione, glutathione-S transferase, superoxide dismutase, and nitric oxide among studied groups.

Group	GSH (mmol/g liver)	GST (U/g liver)	SOD (U/g liver)	NO (μmol/g liver)
**Noninfected untreated**	0.84±0.03	1.44±0.06	1436.9±26.6	285.7±48
**Noninfected+ ZLE therapy**	0.87±0.02	1.49±0.02	1469.8±38.2	288.6±38.9
**P1**	**0.02**	**0.02**	**0.04**	0.88
**Infected untreated**	0.41±0.07	0.81±0.02	859.8±3.5	862.3±86.8
**P2**	**< 0.001**	**< 0.001**	**< 0.001**	**< 0.001**
**Infected + PZQ therapy**	0.61±0.04	1.1±0.14	899.8±12.1	443.2±22.3
**P3**	**< 0.001**	**< 0.001**	**< 0.001**	**< 0.001**
**Infected +ZLE therapy**	0.70±0.07	1.22±0.08	928.6±6.4	373±25.3
**P4**	**< 0.001**	**0.002**	**< 0.001**	**0.002**
**P5**	**0.012**	**< 0.001**	**0.006**	**0.02**

GSH, reduced glutathione; GST, glutathione-S transferase; SOD, superoxide dismutase; NO, nitric oxide; P1 between noninfected untreated and noninfected +ZLE therapy, p2 between noninfected untreated and infected untreated, p3 between noninfected untreated and infected + PZQ therapy, p4 between noninfected untreated and infected + ZLE therapy, p5 between infected + PZQ therapy and infected +ZLE therapy

Some antioxidant parameters (GSH, GST, and SOD) involved in the down-regulation of NO during oxidative stress, have been evaluated in hepatic homogenates. It was amazing that the level of these substances significantly decreased after *S*. *mansoni* infection (p< 0.001), whereas PZQ and ZLE therapy significantly boosted their level (p < 0.05). However, ZLE therapy significantly increases the antioxidant parameters in hepatic homogenates compared to PZQ therapy (p = 0.012, <0.001, and 0.006 respectively) (Table 3 and S3B Fig) and this indicated that ZLE has a powerful antioxidant effect.

### 3.4 Impact on hepatic injury parameters

PZQ and ZLE treatment’s effect on the liver injury parameters of hamsters infected with *S*. *mansoni* was evaluated. Infected untreated hamsters (group III) had significantly higher serum levels of ALT, AST, and alkaline phosphatase compared to group I (noninfected untreated) (p < 0.001) and were significantly lowered with ZLE treatment compared to PZQ treatment (p = 0.004, **<** 0.001, and 0.012 respectively**)**. Therefore, ZLE therapy could have a hepatoprotective effect evidenced by decreasing the hamsters’ hepatic enzymes level (Table 4 and S4 Fig).

**Table 4:** The effect of ZLE and Praziquantel treatment on liver injury parameters in studied groups.

Group	ALT U/L	AST U/L	Alkaline phosphatase IU/L
**Noninfected untreated**	30±2.9	34.3±0.95	60.8±0.6
**Noninfected+ ZLE therapy**	31.2±3.6	37.2±0.6	63.5±0.8
**P1**	1	0.09	0.18
**Infected untreated**	80.7±2.1	97±3.4	123.4±1.3
**P2**	**< 0.001**	**< 0.001**	**< 0.001**
**Infected + PZQ therapy**	43.5±3.1	45.6±3.6	68.7±5.1
**P3**	**< 0.001**	**< 0.001**	**< 0.001**
**Infected +ZLE therapy**	38.8±1.4	40.8±1.9	64.8±1.8
**P4**	**< 0.001**	**< 0.001**	**0.008**
**P5**	**0.004**	**< 0.001**	**0.012**

ALT, alanine aminotransferase; AST, aspartate aminotransferase; P1 between Controls (noninfected untreated) and noninfected +ZLE, p2 between controls and infected untreated, p3 between controls and infected + PZQ, p4 between controls and infected + ZLE, p5 between infected + PZQ and infected +ZLE

## 4. Discussion

Schistosomiasis is a widespread chronic neglected tropical disease. The main challenge in this disease is the lack of successful preventive measures, the increase in the prevalence of PZQ resistance, the shortage of alternative effective drugs, and no treatment for liver fibrosis. Numerous in *vivo*/in *vitro* studies evaluated the anti-*S*. *mansoni* efficacy of raw plant extracts to solve these problems and create novel medication (s) [28,29].

Angiogenesis plays a crucial part in the development and progression of hepatic fibroproliferative and ischemic disorders including hepatic schistosomiasis. The main pro-angiogenic factor both in vivo and in vitro is vascular endothelial growth factor (VEGF). Liver diseases cause inflammation and hypoxia, increasing VEGF levels. VEGF may be used to predict the outcome of the liver and to assess the therapeutic response of these patients [30–32].

Since there is very little research about the effect of ZLE on Schistosomal hepatic fibrosis, to our knowledge, our study is the first study that explored the anti-angiogenic activity and anti-proliferative activity of ZLE as a possible mechanism for reducing fibrosis in this context. Therefore, we aimed to evaluate the therapeutic potential of ZLE as an anti-angiogenic, and anti-proliferative agent as a promising hepatoprotective therapy against *S*. *mansoni-*induced liver fibrosis in hamsters.

In this work, *S mansoni* -infected untreated hamsters overexpressed the hepatic VEGF evidenced by very strong VEGF immunostaining within granulomas compared to control hamsters. Interestingly, we found treatment with ZLE resulted in a negative expression of VEGF in the hepatic tissue compared to both infected untreated and infected treated with PZQ and this confirms the potent anti-angiogenic effect of ZLE.

There is no previous study that explored the anti-angiogenic of ZLE in *Schistosoma*-*mansoni-*induced liver fibrosis to compare our results with. However, Abu Raghif et al., found that methanol ZLE had a strong antiangiogenic impact on rat aorta [32]. Additionally, others demonstrated that PZQ-treated animals had lower levels of VEGF expression [33]. This interesting result may indicate that ZLE therapy might have encouraging effects against conditions linked to angiogenesis. The antiangiogenic properties of ZLE could be attributed to the plant’s multi-biologically active components, including flavonoids which exhibit substantial antiangiogenic effects via modulating the expression of VEGF, MMPs, and EGFR, as well as inhibiting the NFB, PI3-K/Akt, and ERK1/2 signaling pathways [34].

Through integrated signaling networks that control the extracellular matrix’s (ECM) deposition, *S*. *mansoni* infection results in liver fibrosis. The strongest fibrogenic cytokine in the liver is TGF-β1, whose expression rises during fibrogenesis and which is the main factor driving HSCs to boost ECM production. However, there is disagreement on the connection between TGF-β1, liver fibrosis, and Schistosoma infection [35–37].

In our work, the infection of hamsters with *S*. *mansoni* demonstrated pronounced expression of TGF-β1 when compared to their corresponding noninfected untreated group. Treatment with ZLE caused a significant reduction in TGF-β1 expression in the liver when compared to the infected untreated group and PZQ-treated hamsters. Therefore, ZLE exhibited a significant anti-fibrotic role in schistosome hepatic fibrosis and ZLE therapy may reduce the transcription of profibrotic genes in the liver of infected hamsters. With *S*. *mansoni*.

The decreased expression of TGF-β1 in the hepatic tissue with ZLE treatment is consistent with earlier research [18,38], ZLE exerts its anti-fibrotic properties by reducing TGF-β1 expression and inducing de novo synthesis of collagen type III. In contrast to our finding, Alves Oliveira et al. have shown that TGF-β1 is strongly unrelated to fibrosis during *S*. *mansoni* infection [35]. According to our immunohistochemistry findings, ZLE treatment has stronger anti-angiogenic and anti-fibrotic effects than PZQ treatment in schistosome hepatic granulomas and fibrosis. The antifibrotic activity of ZLE is due to a combination of biologically active compounds found in Ziziphus spina-christi, including polyphenols and flavonoids. Polyphenols inhibit fibrosis via inducing apoptosis of activated HSCs, which is predominantly related to NF-*κ*B and TNF—*α* signaling. Furthermore, flavonoids may inhibit HSC proliferation and the expression of profibrogenesis-related genes in HSCs. Via the TGF- *β*1/Smad pathway [39,40].

A combination of clinical evidence and animal studies suggests that schistosomiasis can trigger hepatocellular carcinogenesis through the activation of c-Jun and STAT3 in response to soluble egg antigens. [41–43]. Hepatic injury caused by schistosomiasis was exacerbated by the accumulation of reactive oxygen species, which activated JAK2 and STAT3 [44].

According to the results of this in vivo study, ZLE extracts have anti-proliferative properties, as evidenced by a decrease in the percentage of hepatocytes positive for Ki-67. Ki-67 expression is significantly linked to tumor cell proliferation and growth. We have found no previous study that evaluated the anti-proliferative activity of ZLE and praziquantel in *Schistosoma-mansoni*-induced liver proliferation. However, Hao et al. demonstrated that praziquantel has an antiproliferative effect during the treatment of psoriasis in mice by inhibiting STAT3 phosphorylation, thereby suppressing Th17 immunity [45]. In addition, many studies highlight the anti-proliferative potential of ZLE in cancer patients. The antiproliferative activity was evaluated in-vitro on cervical OV2008, breast MCF-7 cancer, C643 thyroid carcinoma cell lines, and cervical (HeLa) cancer cell lines [46,47]. The presence of polyphenols in ZLE contributes to its antiproliferative activities [39].

Numerous studies have linked ROS to the induction of angiogenesis and fibrogenesis [48,49]. As a result of Schistosomiasis infection, free radicals are released and the antioxidant system in the cell is disrupted. Due to the increased formation of reactive oxygen intermediates, it is recognized that oxidative stress mechanisms are crucial in mediating liver damage in schistosomiasis. *Schistosoma mansoni* infection may unbalance oxidative parameters through a variety of processes, including egg deposition, changes in vascular tone, and soluble immune intermediates [50,51].

In our work, there is a highly significant rise in NO in hepatic homogenates and a decrease in antioxidant markers (GSH, GST, SOD) in *S*. *mansoni-*infected hamsters compared to controls and this suggests that schistosomiasis results in a greater release of free radicals and the increase in NO, indicates excessive oxidant generation. Decreasing GST and SOD activity as well as depletion of non-enzymatic antioxidants like GSH may be linked to the increased oxidative stress and liver cytotoxicity of increased NO levels as the antioxidant pathways are probably unable to compensate for the increase in oxidant production. The presence of granuloma and immune-related cell types may be the primary cause of the increase in oxidative stress in the hepatic tissue, as the immunological response of inflammatory cells is known to be associated with the formation of reactive oxygen species and oxidative injury [52].

ZLE treatment greatly lowered the rise in NO caused by *S*. *mansoni* and increased antioxidant parameters indicating that ZLE prevents oxidative stress and maintains the antioxidant capacity of the liver. This effect might be correlated to the ability of ZLE to kill immature worms and avoid egg deposition in the liver. The potent antioxidant activity of ZLE may be related to the presence of different polyphenols in the extract. According to Nabavi et al., polyphenols can reduce oxidative stress and inflammation by targeting Nrf2 and subsequently activating the cytoprotective genes connected to the antioxidant response element [53]. Therefore, our results suggest that ZLE therapy exhibits potent antioxidant properties.

Our study reinforces the previous studies that reported the administration of ZLE for hamsters experimentally infected with *S*. *mansoni* have a pronounced antischistosomal effect evidenced by a significant decline in the hepatic tissue egg load and a significant reduction in the size and number of granulomas compared to infected untreated hamsters [18,54].

In schistosomiasis, the Th1/Th2 responses play a crucial role in controlling granulomatous inflammation and liver fibrosis. Animal studies suggest that liver fibrosis and granuloma formation are mediated by a persistent and dominant Th2 response brought on by egg-derived antigens [55,56].

The reduction of granuloma size and granuloma area by ZLE in our study may be related to its role in the upregulation of Th1 interferon-gamma cytokine and declines of Th-2 mediated cytokine IL-4 which leads to a reduction of granuloma size [56].

Several plant extract compounds are shown to have schistosomicidal effects, prevent ROS formation, and possess anti-inflammatory activities.

Administration of *S*. *nigrum* leaves extract and R. vomitoria stem and root extract have antischistosomal effect proven by significant reduction in the number of worms per infected mouse [57,58]. Moreover, leaves extract of *Z*. *officinale* and *C*. *umbellatum* were reported to have a schistosomicidal, antioxidant effect. Their antioxidant properties enabled them to scavenge free radicals and restore normal hepatocytes and hepatic strand organization [59,60]. Ginger loaded on chitosan NPs have a potential anti-schistosomal effect, alleviating the inflammatory response of the host [61].

Compared with other plant extracts ZLE have been proved to possess antiproliferative, anti- angiogenesis, anti-fibrotic effect besides its schistosomicidal and antioxidant effect.

The histopathological examination of the livers in the PZQ-treated group revealed a significant reduction in the diameter of liver eggs granuloma and granuloma area compared to the infected untreated group. These results are correlated to El-Lakkany and Nosseir [62]. It has been suggested that PZQ has strong regulatory effects on cell immunological responses, reducing CD4+T cells while increasing CD8+ cells, resulting in a smaller hepatic granuloma size in schistosomiasis [63].

It is known that *S*. *mansoni* infection causes hepatocellular injury, which then triggers the release of enzymes from the damaged hepatic cells into the bloodstream [64].

In the current study, ZLE treatmen”s effect on the liver injury parameters of hamsters infected with *S*. *mansoni* was evaluated. Infected untreated hamsters had significantly higher serum levels of ALT, AST, and alkaline phosphatase compared to noninfected untreated and were significantly lowered with ZLE treatment compared to PZQ treatment. The improvement of liver enzymes may be related to improvement in the histological activity index, and anti-inflammatory and anti-oxidative properties of ZLE.

The presence of inflammatory hepatic granuloma that is present as a result of egg deposition and its toxins may be the cause of the high levels of liver enzymes. Serum transaminases have been found to rise in *S*. *mansoni-*infected mice by another researcher [65]. Therefore, ZLE therapy could have a hepatoprotective effect evidenced by decreasing the hamster” hepatic enzymes level.

The preferred and the most effective medication for treating human schistosomiasis is PZQ. It has been documented that certain strains of *S*. *mansoni* and *S*. *japonicum* are resistant to praziquantel in the laboratory, as well as in many endemic foci of *S*. *mansoni*, as in Egypt and Senegal. This is due to drug pressure on praziquantel for numerous decades as a sole medication and its successive uses in the treatment of the infected vector snail, *Biomphalaria glabrata* [66–67]. Consequently, researchers have increased their efforts to research alternative drugs for treating schistosomiasis.

PZ”s acute toxicity is relatively low, as demonstrated by an intraperitoneal LD50 value 564 mg/kg and intragastric LD50 value of 2,976 mg/kg in various species [68]. Dhuha et al., on the other hand, demonstrated that ZLE had a rather high hazardous dosage, namely 4050 mg/kg [69].

The *Ziziphus spina-christi* plant is considered a valuable tree due to its active components, safety, and ease of cultivation in the future [70].

According to the current study, these results collectively suggest that ZLE therap”s anti-angiogenic, anti-proliferative, and antifibrogenic properties, as well as the live”s lowered egg burden and granuloma width, may be responsible for the therap”s reduced hepatic damage parameters and this is reflected in an improvement of the liver enzymes and this established the hepatoprotective effects of ZLE treatment.

## 5. Limitation of the study

More research is needed to investigate the efficacy of different doses of ZLE and the potential synergistic effect of both PZQ and ZLE in a model of *S*. *mansoni* hepatic fibrosis. The toxicity of ZLE should be monitored beyond 48 hours of gavage.

## 6. Conclusion

In conclusion, the methanol ZLE showed not only an anti-fibrinogenic effect but also a prominent anti-angiogenic and antiproliferative effect on hepatic *S*. *mansoni* granulomas compared to PZQ and this activity may be due to the high free radical scavenging capacity. So, ZLE therapy may have promising activity against angiogenesis, and proliferative-related hepatic diseases. More research is still required to clarify its safety in people and thoroughly explore any potential therapeutic effects.

## Supporting information

S1 Fig The mean granuloma area in hamsters treated with both PZQ and ZLE was significantly reduced compared to infected untreated hamsters (p = 0.001, < 0.001 respectively). (TIF)

S2 Fig Treatment of infected hamsters with PZQ and ZLE significantly reduce the mean number of worms compared to the infected untreated group (p < 0.001 for all). (TIF)

S3 Fig A) Treatment with PZQ and ZLE highly significantly reduced the NO levels in hepatic homogenates (p < 0.001 for both), however, ZLE therapy significantly reduce the NO in hepatic homogenates compared to PZQ therapy (p = 0.02). (B) ZLE therapy significantly increases the SOD in hepatic homogenates compared to PZQ therapy (0.006 respectively).
(TIF)

S4 Fig Infected untreated hamsters had significantly higher serum levels of ALT compared to noninfected untreated (p < 0.001) The ALT was significantly lowered with ZLE treatment compared to PZQ treatment (p = 0.004). (TIF)

S1 Data Supporting data for figures and tables. (XLSX)
