# Supplementary figures and images for: Anti-angiogenic and anti-proliferative activity of ziziphus leaf extract as a novel potential therapeutic agent for reducing hepatic injury in experimental hamster schistosomiasis
Source: PLoS Negl Trop Dis. 2023 Jun 20;17(6):e0011426. doi: 10.1371/journal.pntd.0011426 (PMC10313029; doi:10.1371/journal.pntd.0011426)

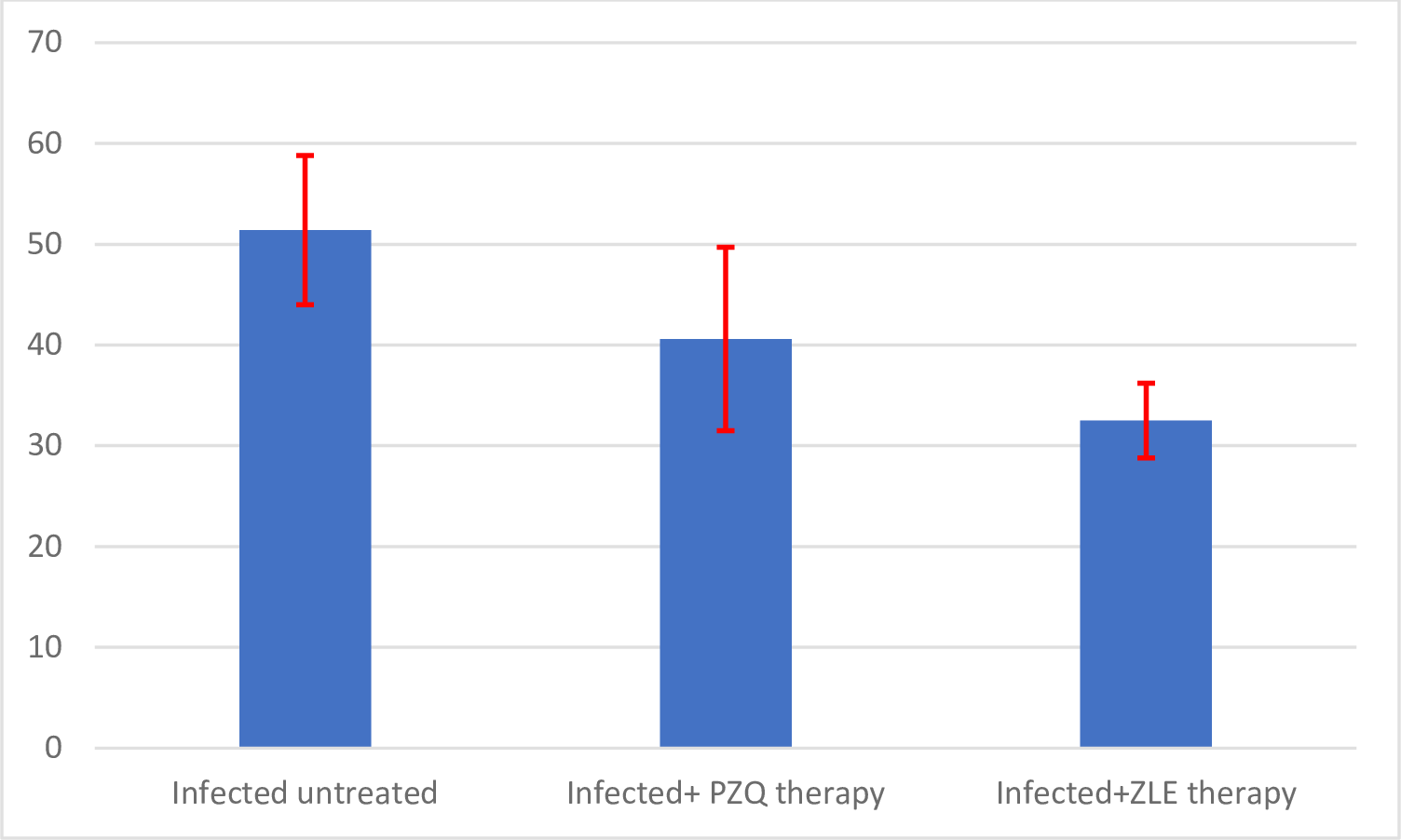

Supplement: S1 Fig — (TIF) [file pntd.0011426.s001.tif]

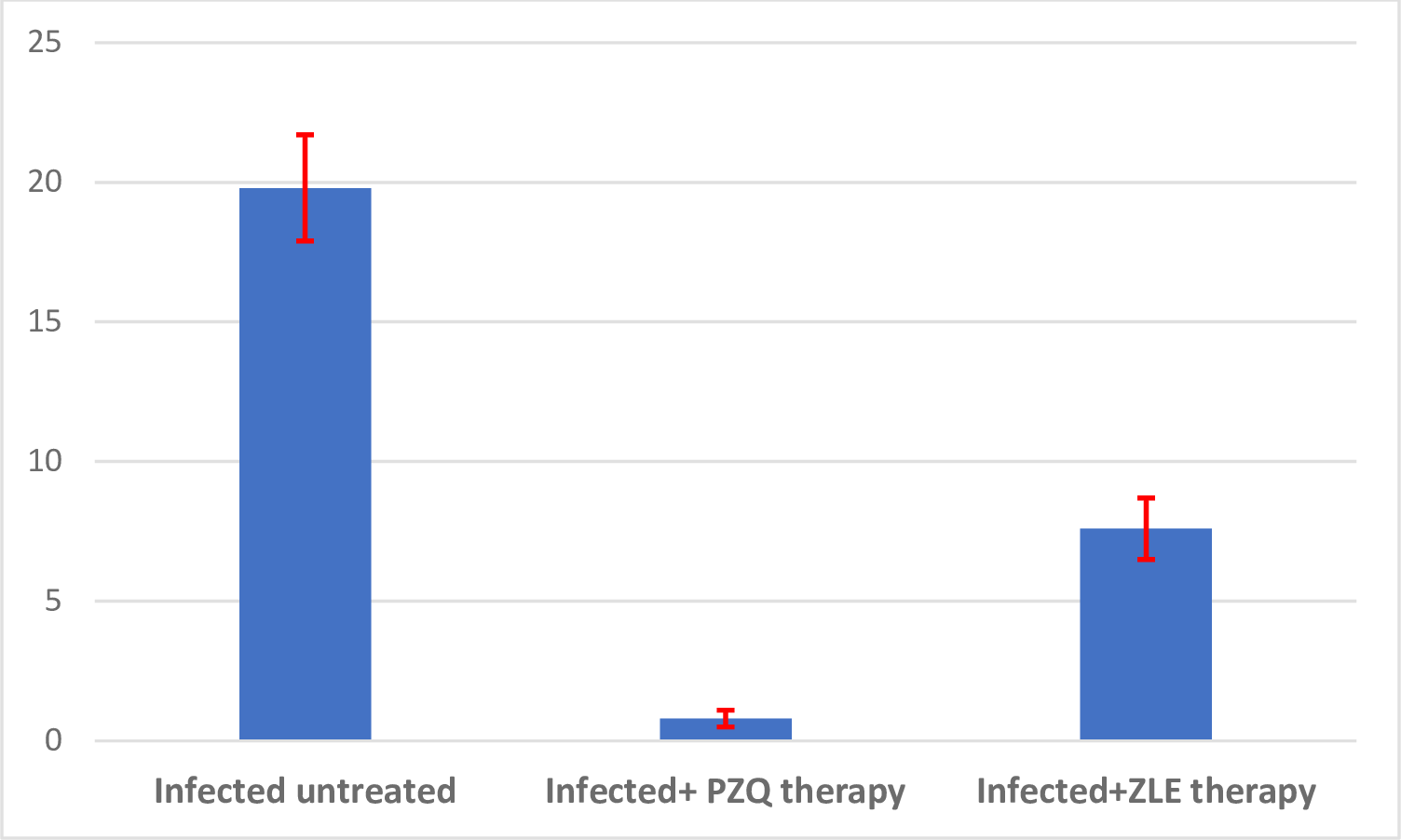

Supplement: S2 Fig — (TIF) [file pntd.0011426.s002.tif]

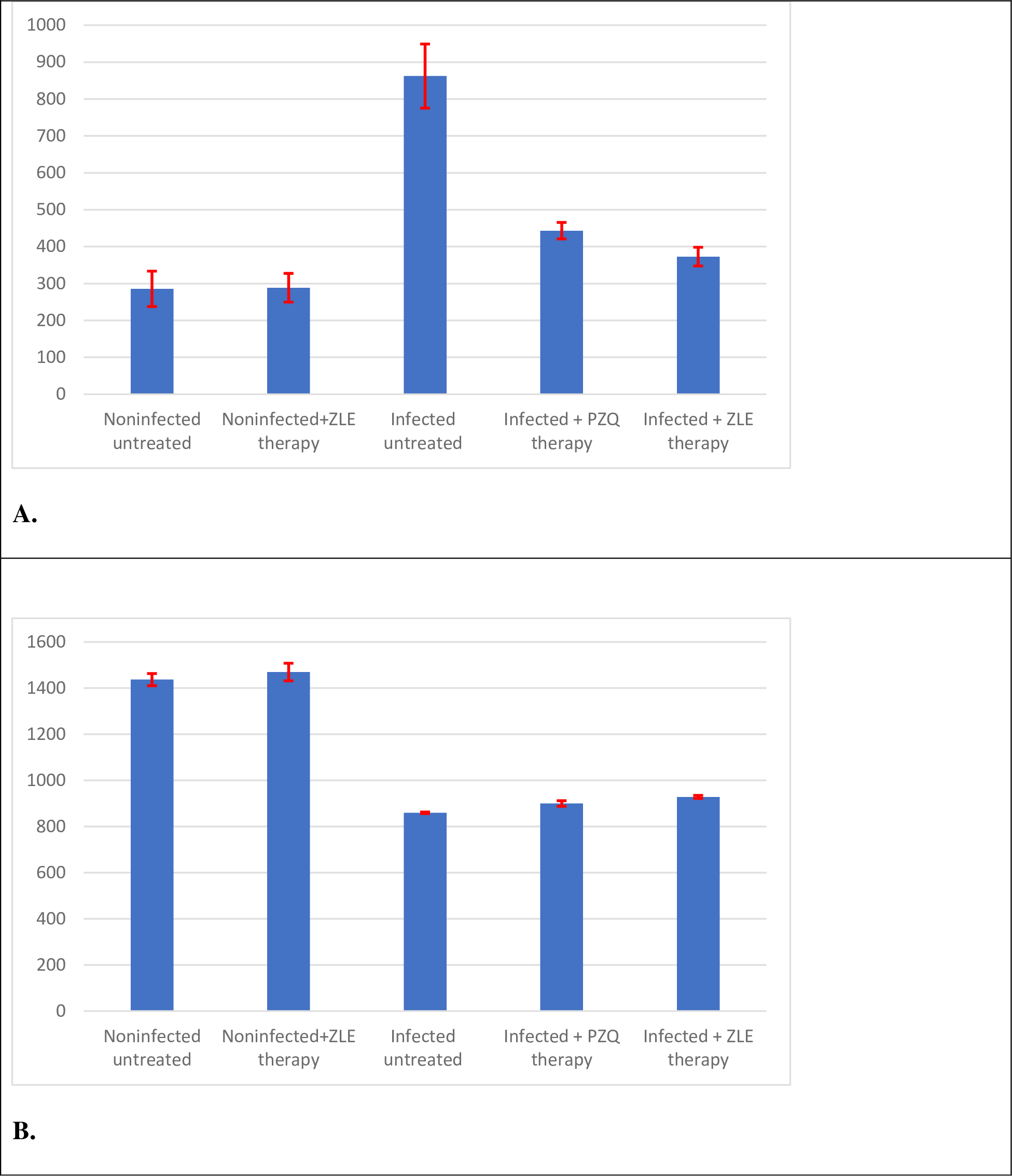

Supplement: S3 Fig — A) Treatment with PZQ and ZLE highly significantly reduced the NO levels in hepatic homogenates (p < 0.001 for both), however, ZLE therapy significantly reduce the NO in hepatic homogenates compared to PZQ therapy (p = 0.02). (B) ZLE therapy significantly increases the SOD in hepatic homogenates compared to PZQ therapy (0.006 respectively). (TIF) [file pntd.0011426.s003.tif]

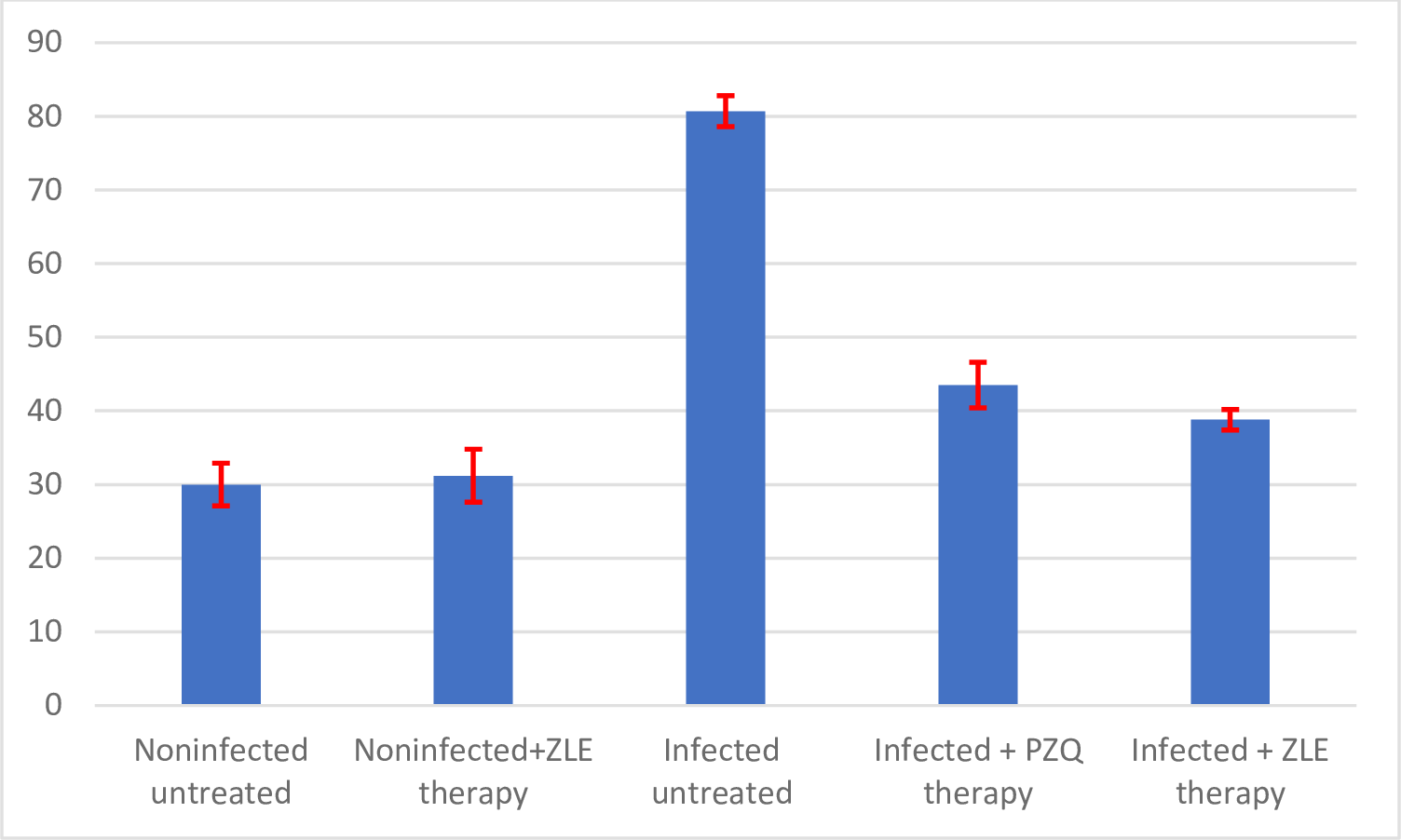

Supplement: S4 Fig — (TIF) [file pntd.0011426.s004.tif]
